# Perceived Intelligence Is Associated with Measured Intelligence in Men but Not Women

**DOI:** 10.1371/journal.pone.0081237

**Published:** 2014-03-20

**Authors:** Karel Kleisner, Veronika Chvátalová, Jaroslav Flegr

**Affiliations:** Department of Philosophy and History of Science, Faculty of Science, Charles University in Prague, Czech Republic; University of Goettingen, Germany

## Abstract

**Background:**

The ability to accurately assess the intelligence of other persons finds its place in everyday social interaction and should have important evolutionary consequences.

**Methodology/Principal Findings:**

We used static facial photographs of 40 men and 40 women to test the relationship between measured IQ, perceived intelligence, and facial shape. Both men and women were able to accurately evaluate the intelligence of men by viewing facial photographs. In addition to general intelligence, figural and fluid intelligence showed a significant relationship with perceived intelligence, but again, only in men. No relationship between perceived intelligence and IQ was found for women. We used geometric morphometrics to determine which facial traits are associated with the perception of intelligence, as well as with intelligence as measured by IQ testing. Faces that are perceived as highly intelligent are rather prolonged with a broader distance between the eyes, a larger nose, a slight upturn to the corners of the mouth, and a sharper, pointing, less rounded chin. By contrast, the perception of lower intelligence is associated with broader, more rounded faces with eyes closer to each other, a shorter nose, declining corners of the mouth, and a rounded and massive chin. By contrast, we found no correlation between morphological traits and real intelligence measured with IQ test, either in men or women.

**Conclusions:**

These results suggest that a perceiver can accurately gauge the real intelligence of men, but not women, by viewing their faces in photographs; however, this estimation is possibly not based on facial shape. Our study revealed no relation between intelligence and either attractiveness or face shape.

## Introduction

The human face is a complex structure with a crucial social signalling function. Though numerous and varied mammalian species exhibit well developed facial structures, the communicative and expressive roles of the face reach a unique level of ability in human beings [1]. It is well established in previous research that faces inform us about personality, sex, age, health, ethnicity, social rank, attractiveness and political affiliation, as well as, to some extent, the intelligence of the bearer [2]–[9].

Some researchers have suggested that people tend to attribute higher intelligence to attractive persons [10], [11]. If women tend to prefer intelligent men because of their generally higher social status and these men in turn tend to prefer attractive women, the alleged covariance of attractiveness and intelligence should be of no surprise [12]. However, such findings are controversial and should be approached cautiously since Kanazawa's research methods and conclusions have attracted strong criticism [13]–[15]. As with physical attractiveness, intelligence is suggested to indicate good genes [16], [17]. This notion is supported by the fact that during the fertile phase of their menstrual cycle, women display a higher preference for men who score highly in creative intelligence [18]. Intelligence is also correlated with humour, which is suggested to have evolved in sexual selection as an intelligence-indicator [19]. By modifying the good genes approach we find a bad genes hypothesis, which argues that even though unattractive faces signal poor genetic fitness, there is no difference in genetic fitness between faces of average and high attractiveness [20].

It has been also suggested that sexual selection has played a role in the evolution of particular facial features, which have evolved to signal high intelligence [10], [21]. Visual cues responsible for a higher attribution of intelligence may honestly reflect the real intelligence of a person and can therefore be used to indicate a preferential sexual or social partner. Past research shows that people are able to judge intelligence from the facial qualities of unknown persons, r = 0.28 [10]. Measured intelligence has been shown to correlate with perceived intelligence and other personality traits, whether self-reported (0.29) or rated either by intimate acquaintances (0.31) or strangers (0.38) [22]. In addition to visual cues, there is evidence of the accurate assessment of intelligence based on behavioural and acoustic cues [23]–[26]. For example, measured intelligence significantly correlates with ratings by stranger in sound-film conditions (0.38) but not in silent-film conditions [22] and the correlation between measured intelligence and ratings of fluent speech is as high as r = 0.53 [23]. In general, inferences of intelligence taken from thin slices of behaviour have been shown to be highly accurate [27].

Gender influences the accuracy of intelligence assessment. Men were more accurately assessed for intelligence than women, while women were more accurate at assessing the intelligence of both men and women [26], [28].

Although a number of studies have examined the perception of intelligence from different visual cues, none of these studies describe the specific facial traits that play a role in intelligence assessment. The specific aim of the present study is to determine which facial shape cues are responsible for the attribution of intelligence, as well as those which correlate with actual intelligence. We also sought to identify which particular factors of general intelligence can accurately be assessed from facial photographs. Finally, using thin-plate spline extrapolation, we provide a statistically supported description of the intelligence-stereotype in order to depict the facial traits responsible for an attribution of intelligence.

## Methods

The present study integrates data from two different studies in which two independent groups of students participated: the first group consisted of 80 biology students at the Faculty of Science who were tested for IQ and photographed; the second group involved 160 raters, students at the Faculty of Humanities who rated the photos of the biology students either for intelligence or for attractiveness (each student rated 80 randomized photos). Dataset is available at http://web.natur.cuni.cz/flegr/IQKleisneretal2013.xls

### Ethics statement

The Institutional Review Board of Charles University, Faculty of Science approved this research. Written informed consent was obtained from all participants involved in our study. The data on measured intelligence, perceived intelligence and attractiveness were analyzed anonymously.

### IQ measurement

**Figure 1:**
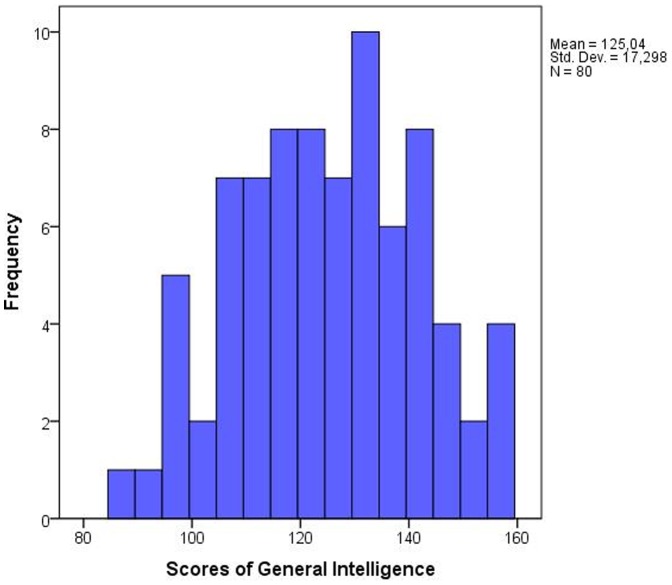
Histogram showing the range of distribution of IQ among university students involved in the present study.

To measure the intelligence of subjects, we used a Czech version of Intelligence Structure Test 2000 R [29], [30]. The test consists of a basic module, which is comprised of three verbal, three numerical, and three abstract figural reasoning tasks. The test also includes two memory tasks and a knowledge test. The knowledge test is focused on questions from geography/history, business, science, mathematics, arts, and daily life. As a whole, the test obtains a broad spectrum of results: the basic module measures verbal, numerical and figural IQ, as well as memory and reasoning; the knowledge test measures verbally, numerically and figurally coded knowledge; and both parts of the test measures fluid, crystallized, and general IQ. Fluid intelligence is the capacity to think logically and solve problems in novel situations, independent of acquired knowledge. This sort of reasoning does not reflect cultural differences but arises from biologically given cognitive abilities. Crystallized intelligence is the ability to use skills, knowledge, and experience. This sort of reasoning improves with age and reflects the lifetime achievement of an individual [31]. Verbal intelligence is the ability to use language to analyze and solve problems associated with language-based reasoning. Numerical intelligence is the ability to manipulate numerical symbols and to comprehend quantitative relationships. Figural intelligence is the ability to combine shapes and forms and to analyze spatial patterns. The university students involved in this study showed a broad range of distribution of IQ (see Fig. 1.)

We administered the exam on computers to 10–12 individuals in the same room and at the same time (at 9:15 am). The total length of time was about 145 minutes, including a15 minute break between the basic module and the second module with the memory tasks and knowledge test.

### The photographs

Facial photographs of 80 students (40 men: Mean± SD = 21.8±2.8, range: 19–34, and 40 women: Mean± SD = 20.9±1.6, range: 19–24) from the Faculty of Science, Charles University in Prague, Czech Republic, were used as stimuli. The subjects were seated in front of a white background and photographed with a digital camera, Canon 450D, using studio electronic flash and reflection screen. The subjects were instructed to adopt a neutral, non-smiling expression and avoid facial cosmetics, jewellery, and other decorations. The photos were cropped to place the eyes horizontally at the same height and leave a standard length of neck visible.

### Rating the photographs

One hundred sixty raters (75 men and 85 women) took part in judging the photographs; they had no connection either to the Faculty of Science or the rated subjects and were aged 26.7 years on average (women: Mean± SD = 26.7±7.7, range: 15–58; men: Mean± SD = 26.7±7.5, range: 16–55). Of these, 43 women (Mean± SD = 27.1±8.8, range: 15–58) and 42 men (Mean± SD = 28.5±8.2, range: 20–55) judged the subjects for intelligence. Another 42 women (Mean± SD = 26.2±6.4, range: 21–51) and 33 men (Mean± SD = 24.5±5.7, range: 16–38) judged the photos for attractiveness. Every person rated the whole set of 80 photos, either for perceived intelligence or attractiveness, using a seven-point scale wherein 1 stands for the highest ranking (for intelligence or attractiveness) and 7 the lowest (intelligence or attractiveness). The presentation and judgment of all photographs were performed using the software ImageRater 1.1.

The raters were individually invited to judge the photographs. Each rater saw the photographs on a computer screen and indicated their valuation by mouse clicks on a discrete seven-point scale. No time limit was imposed. The order of the photographs was randomized for each rater. In the situation where a rater knew or was acquainted with a person pictured, she/he was instructed not to rate that picture. To eliminate the influence of individual differences between raters, the ratings of all photographs evaluated by each rater were converted to z-scores and the perceived intelligence/attractiveness of each photographed subject was calculated as its average z-score. The z-scores of perceived intelligence and attractiveness ratings were normally distributed.

### Statistics

The relationship between measured IQ and perceived intelligence was tested by linear regression models using a mean z-score of perceived intelligence as the dependent variable and IQ values as the independent variable. The age of photographed individuals and raters was added as a covariate. We measured a Pearson correlation between perceived intelligence and perceived attractiveness to estimate the intensity of the halo effect in our population. Perceived attractiveness was added to the model as a covariate, because perceived intelligence strongly correlates with attractiveness. Each of the intelligence components was tested separately for men and women. Both quadratic and linear models were fitted, their statistical plausibility evaluated by F-test and the Akaike Information Criterion (AIC). The effect size of an explanatory variable was expressed by partial η^2^. Because the averaging of individual ratings can inflate the effect size of correlations [32], we have also calculated partial Pearson correlation between IQ and perceived intelligence for each rater with perceived attractiveness as a covariate and calculated average partial Pearson correlation for all raters. Significance level was estimated by permutation test with 10,000 randomizations. For each randomization, the order of values, represented by raters' assessment of perceived intelligence for 40 photos, was randomly changed. The partial Pearson correlation coefficient between permuted judgments of each rater and IQ values of photos was then computed and these correlation coefficients were averaged for all raters. A comparison of average correlation coefficient computed with original data with average coefficients computed from randomized sets of data (namely the fraction of higher or equal coefficients computed from randomized data sets) provided the statistical significance of the permutation test. For statistical analyses we used PASW/SPSS 18 and R statistical software version 2.13.2. For the permutation test we used Matlab v. 7.10.0.499.

### Geometric morphometrics

Photographs of 40 men and 40 women were analyzed by geometric morphometric methods (GMM) in order to detect the facial features that are associated with either or both the perception of intelligence and intelligence measured with the Intelligence Structure Test in both men and women.

The 72 landmarks (including 36 semilandmarks) were digitized by tpsDig2 software, ver. 2.14 [33]. Landmarks are represented as points that are anatomically (or at least geometrically) homologous in different individuals, while semilandmarks serve to denote curves and outlines. The definitions of landmark and semilandmark locations on human faces were derived from previous work [34]–[37]. Semilandmarks were slid by tpsRelw (ver. 1.49) software. All configurations of landmarks and semilandmarks were superimposed by generalized Procrustes analysis (GPA), implemented in tpsRelw, ver. 1.46 [33]. This procedure standardized the size of the objects and optimized their rotation and translation so that the distances between corresponding landmarks were minimized. To observe the variation among the landmark data configurations of all specimens, the principal component analysis (PCA) – i.e., the relative warp analysis for parameter α = 0 – was carried out in tpsRelw, ver. 1.46. [33]. To observe the shape variation associated with perceived intelligence/IQ, we regressed GPA shape coordinates onto scores of intelligence rating/IQ by using a multivariate regression in which the dependent variable was the shape coordinates and the independent variable was perceived intelligence ratings or IQ scores; this was conducted in tpsRegr, ver. 1.36 [38]. Shape regressions were displayed by thin-plate splines as a deformation from the overall mean configuration (the consensus) of landmarks. The composite images were constructed by tpsSuper 1.14 [39] using the original photographs of men and women that were unwarped to fixed configuration represented by the estimates of shape regressions.

## Results

### Are intelligent people perceived as more intelligent?

We found a positive correlation between perceived intelligence and perceived attractiveness: r = 0.762, N = 80, p<0.001. This correlation was much stronger in the judgment of women's faces (r = 0.901, N = 40, p<0.001) than those of men (r = 0.502, N = 40, p<0.001). This difference was statistically significant (*z = 3.98, p<0.001*). The ratings for intelligence done by both men and women were highly correlated (r = 0.88, p<0.001), therefore we decided to analyse the ratings of both sexes together.

Linear regression was used to test for an association between perceived intelligence and IQ. We built linear and quadratic models with perceived intelligence as the dependent variable and measurement of IQ as the independent variable. Age, sex, attractiveness rating, and interaction between sex and attractiveness were added as covariates in the models. The quadratic model was statistically preferred over the linear model (F_1,73_ = 4.21, p_(quadratic>linear)_ = 0.043).

Akaike information criterion also preferred the quadratic model (AIC_quadratic_ = −9.2321) to the linear one (AIC_linear_ = −6.748). In accordance with these criteria, we applied the better-fitted quadratic model to analyze our dataset. The effect of interaction between sex and attractiveness was significant (p = 0.006), therefore we ran all these analyses separately for men and women.

IQ significantly affects the perception of intelligence in men (F_1,35_ = 8.13, p = 0.007, η^2^ = 0.188), but there was no such effect for women (F_1,36_ = 1.345, p = 0.254, η^2^ = 0.036). The positive effect of age was significant for men (p>0.001) but not for women (p = 0.274). The effect of perceived attractiveness was significant for both men (p<0.001) and women (p<0.001). Each of the intelligence components was analyzed separately; this showed a significant relationship of perceived intelligence, though only with Figural and Fluid intelligence and only in men (see Table 1).

**Table 1:** Linear and quadratic relationships between IQ and perceived intelligence.

	Men				Women			
	Linear		Quadratic		Linear		Quadratic	
IQ	p-value	partial η^2^	p-value	partial η^2^	p-value	partial η^2^	p-value	partial η^2^
General	0.005**	0.203	0.007**	0.0188	0.935	>0.001	0.866	0.001
Verbal	0.801	0.002	0.810	0.002	0.069	0.091	0.073	0.089
Numerical	0.16	0.056	0.206	0.045	0.807	0.002	0.853	0.001
Figural	0.003**	0.023	0.005**	0.206	0.780	0.002	0.656	0.006
Crystallized	0.188	0.049	0.171	0.053	0.693	0.005	0.623	0.007
Fluid	0.016*	0.156	0.023*	0.139	0.764	0.003	0.828	0.001

* significance level <0.05 (two-tailed).

** significance level <0.01 (two-tailed).

General and figural intelligence of men remained statistically significant (<0.05) after Bonferroni correction. Fluid intelligence was not statistically significant (>0.05) after Bonferroni correction.

When the individual ratings of perceived intelligence were correlated with IQ (general intelligence) with perceived attractiveness as a covariate, the average partial correlation (men: r = 0.061, women: r = 0.023) was significant on the basis of permutation test for men (p<0.001) but not for women (p = 0.09).

### Are intelligent people more attractive?

To test the effect of intelligence on attractiveness, we regressed the attractiveness ratings on the IQ scores for general intelligence. The age of the photographed subject was added to the model as covariate. We found no effect of IQ on perceived attractiveness, either for men (F_1,37_ = 1.748, p = 0.139, η^2^ = 0.045) or for women (F_1,37_ = 0.346, p = 0.346, η^2^ = 0.024). The effect of age was not significant for women (p = 0.99) but close to significant for men (p = 0.062).

### Shape space of perceived intelligence and IQ

We found no relationship between facial morphospace and IQ as measured by IQ test. The regression of shape data on IQ showed no significant result; Goodall's permutation F-test for 5000 iteration showed p = 0.310 for men, and p = 0.895 for women. When particular intelligence components were regressed individually on shape data, no results were statistically significant: Verbal Intelligence for men p = 0.424, women p = 0.906; Numerical Intelligence, men p = 0.352, women p = 0.535; Figural Intelligence, men p = 0.283, women p = 0.950; Crystallized Intelligence, men p = 0.526, women p = 0.345; Fluid Intelligence, men p = 0.301, women p = 0.892 – all permuted by 5000 iterations.

**Figure 2:**
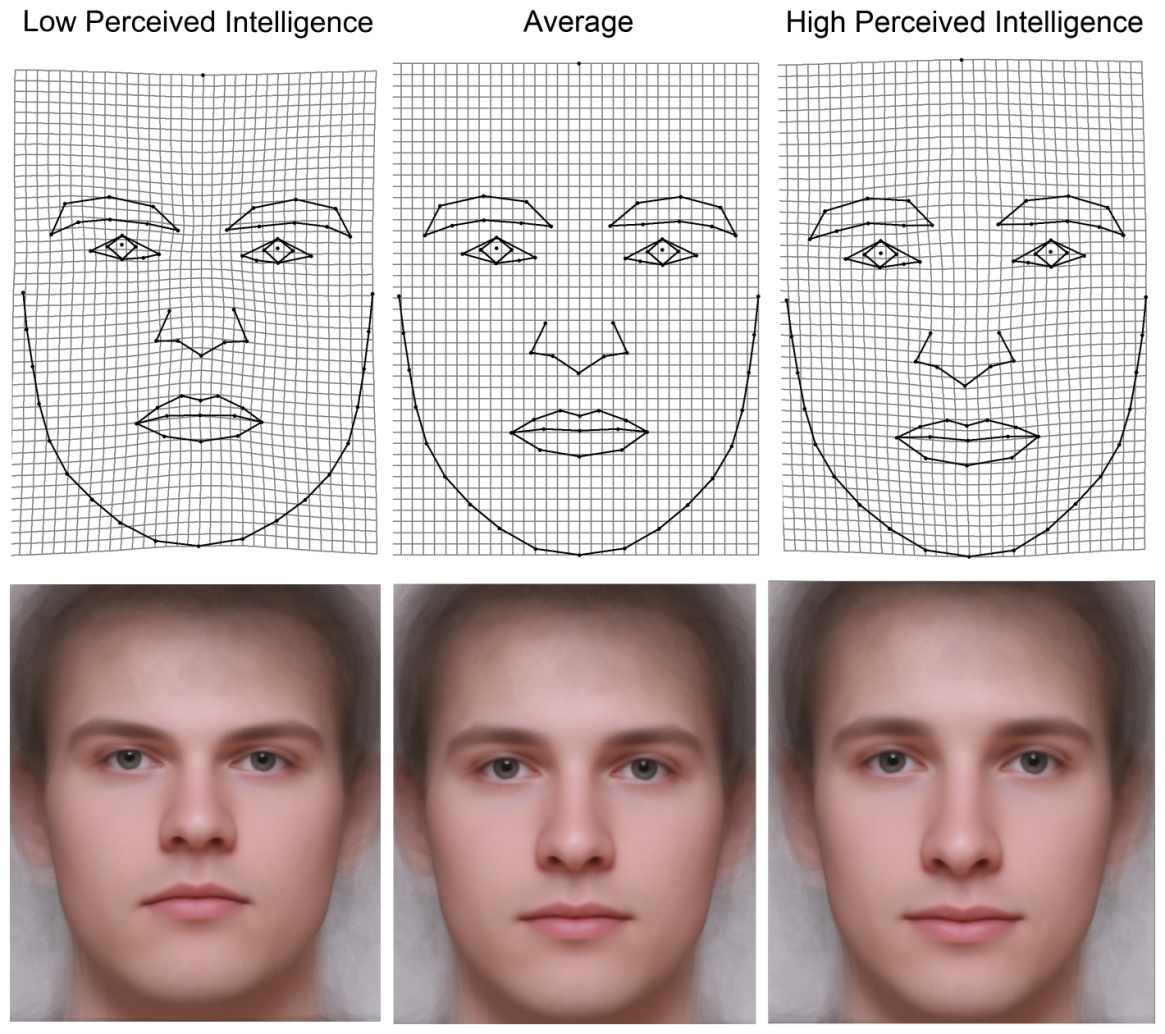
Shape regression on perceived intelligence in men represented by thin-plate spline deformation grids showing differences in facial shape between faces with attributed high intelligence (upper left) and low intelligence (upper right) compared to an average configuration in the middle. The lower panel shows composite images of 40 men photographs unwarped to the fixed landmark configuration predicted by shape regression (each composite corresponds to a particular TPS grid above). The predictions are not magnified by any factor and match the observed range.

**Figure 3:**
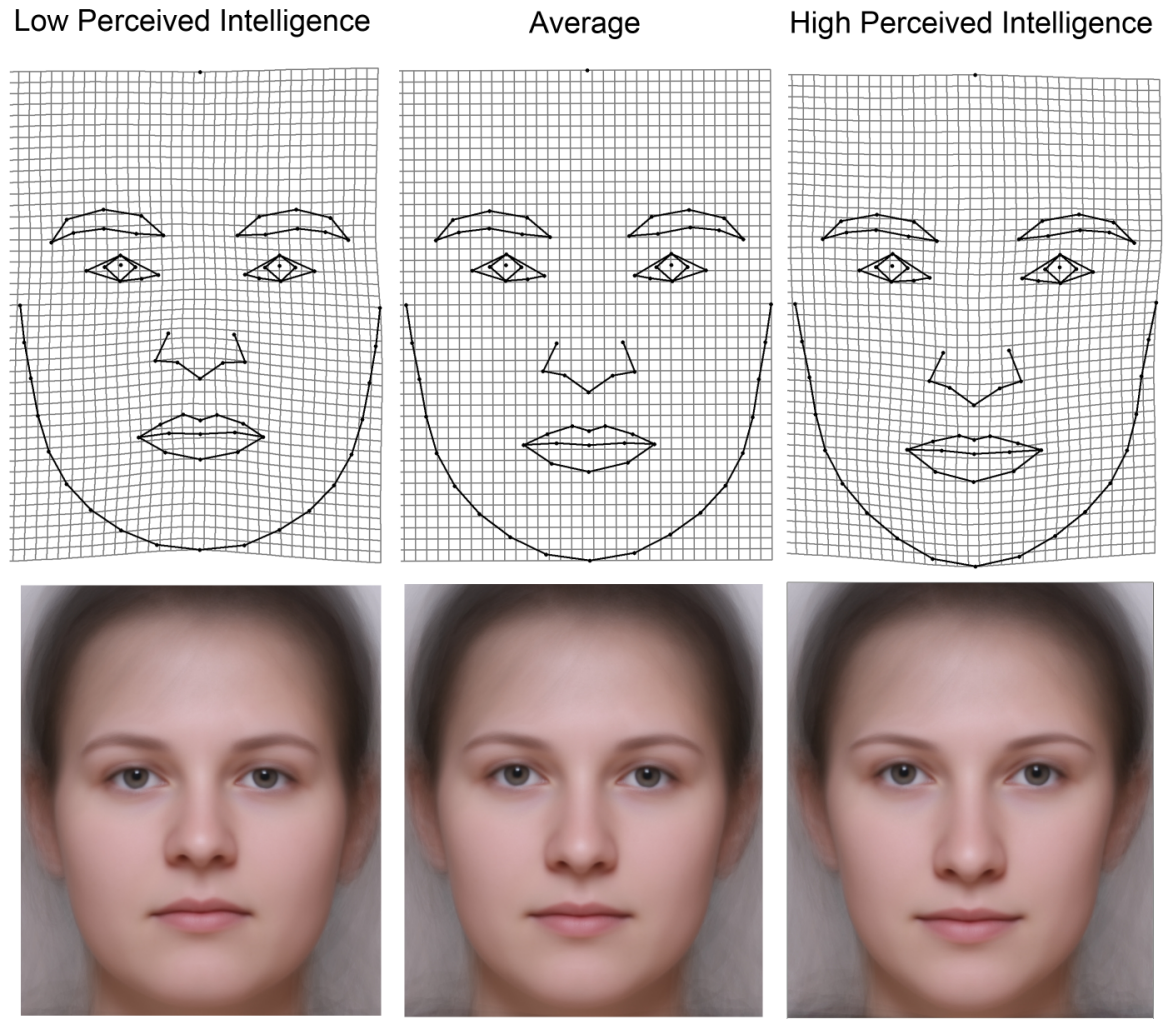
Visualizations of shape regression on perceived intelligence in women by thin-plate spline deformation grids illustrating shape differences between faces with attributed high intelligence (upper left) and low intelligence (upper right) compared to an average configuration in the middle. The lower panel shows composite images of 40 women photographs unwarped to the fixed landmark configuration predicted by shape regression (each composite corresponds to a particular TPS grid above). The predictions match the observed range.

Nevertheless, the shape regression showed a significant relation between perceived intelligence and facial shape for both men and women: Goodall's permutation F-test for 5000 iteration showed p = 0.0052 for men and p = 0.0024 for women. Faces that garner a higher attribution of intelligence show overall dilations of TPS deformation grid in the area between the eyes and mouth. Further grid deformations cover the distance between the eyebrows, an enlargement at the root of the nose, and a markedly prolonged nose. The area of the chin tends to be constricted. By contrast, faces with a lower attribution of intelligence are characterized by constriction in the area between the mouth and eyes, eyebrows closer to each other, the base of the nose is rather narrowed, the nose is shorter, and the area of the chin is strongly dilated. The TPS grids for both sexes show the same vector of shape changes (see Figs. 2 and 3); the most apparent difference between the sexes is that grid constrictions/dilations in the area around the eyebrows as well as the base of the nose is more noticeable between high and low intelligent-looking men than in women.

## Discussion

Our raters were able to estimate intelligence with an accuracy higher then chance from static facial photographs of men but not from photos of women. At the same time, we found no differences in the abilities of men and women to assess intelligence from static facial photos: the ratings of both sexes were highly correlated, r = 0.88. Perceived intelligence positively correlated with attractiveness in both men and women. Even though this contrasts some previous findings [10], [11], [40], [41], we did not observe any significant correlation between measured IQ and attractiveness. However, it should be noted that evidence for a relationship between actual intelligence and physical attractiveness in adults seems rather weak. Zebrowitz et al [10] did not find any significant correlation between perceived intelligence and attractiveness in individuals older than 18 years, i.e. middle adulthood (30 to 40) and later adulthood (52 to 60); however Zebrowitz et al did not examine individuals of a similar age bracket to our study (21.4 years in average). The existence of a correlation between attractiveness and intelligence in the age cohort from 19 to 29 years would appear questionable at the very least. Moreover, meta-analytic study has shown that physical attractiveness is not correlated to actual intellectual competence in adults, but is modestly correlated in children [15]. Similarly, other studies have reported close to zero correlation between attractiveness and actual intelligence in adults [40], [41].

We also showed that IQ has no statistically significant association in facial morphology in both men and women (at least as it was delimited by the landmark configuration used within this study). This means that our raters accurately assessed intelligence from faces of men based on visual cues that simply are not explicable from shape variability in men's faces. It is important to recall that our subjects were prompted to assume a neutral expression while their photo was taken and only photos of subjects with a neutral expression were included in the study. We can speculate about attributions of intelligence based on particular configurations of eyes or gaze, colour of eyes, hair and skin, or skin texture. These hypotheses should be tested in future studies.

### Intelligence stereotype

Though we were not able to objectively detect an association between IQ and facial morphology, we can provide a statistically supported description of the stereotype of an intelligent-looking face. Our data suggest that a clear mental image how a smart face should look does exist for both men and women within the community of human raters. The intelligence-stereotype shows the same transformations in facial shape space for both men and women. In both sexes, a narrower face with a thinner chin and a larger prolonged nose characterizes the predicted stereotype of high-intelligence, while a rather oval and broader face with a massive chin and a smallish nose characterizes the prediction of low-intelligence (see Figs. 2 and 3). There also seems to be a correlation between semblances of emotions of joy or anger in perceptions of high or low intelligence in faces, respectively. The ‘high intelligence’ faces appear to be smiling more than the ‘low intelligence’ faces. A similar pattern was described for the perception of trustworthiness [37]. Perceived intelligence correlates with perceived trustworthiness and happiness. Conversely, low-intelligence faces are perceived as untrustworthy and considered angrier [42]. Moreover, perceived intelligence was also shown to be positively associated with perceived friendliness and sense of humour in both male and female faces but negatively related with perceived dominance in faces of women [43].

The face shape associated with a higher perception of intelligence also shows the characteristics of higher perception of attractiveness, while the face shape associated with a lower perception of intelligence shows traits of higher perceived dominance. The positive correlation with attractiveness and trustworthiness and negative correlation with dominance may explain the attribution of higher intelligence to longer, narrower faces.

### Correlation between IQ and perceived intelligence

Two factors of general intelligence were significantly associated with perceived intelligence from men's faces: fluid intelligence and figural intelligence. Fluid intelligence is the capacity to logically solve problems independent of acquired knowledge [31]. It depends on a subject's genetic qualities and, largely, cannot be influenced by continuous exercise or life experience. Figural intelligence describes the ability to handle objects such as images, patterns, and shapes. One cannot explicitly say that figural intelligence is independent of experience acquired during life, though the nature of this ability would also be affected by heritable genetic cues (as well as nurturing or other environmental effects). By contrast, verbal and crystallized intelligence largely depend on the social environment.

**Figure 4:**
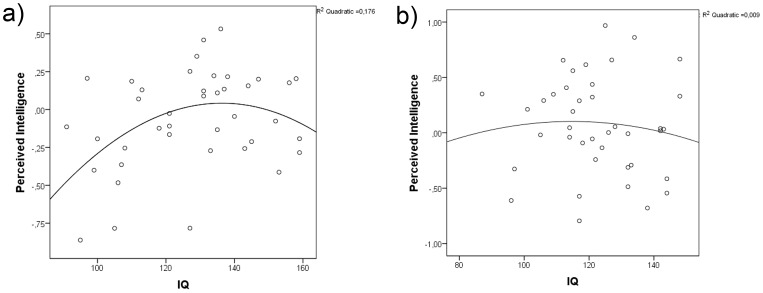
Graph demonstrating linear positive and quadratic negative relationship between IQ and perceived intelligence in men (a) and women (b).

The quadratic shape of the negative relationship between perceived intelligence and IQ points at the constraints that limit the preference of men with a very high IQ. Men with an IQ higher than 140 are perceived as relatively less intelligent (see Fig. 4.), which can reflect an adaptation to an upper intelligence limit as men with an extremely high IQ could find such practical tasks, such as the care and protection of women and offspring, less rewarding. Though intelligence does not positively correlate with mental disorders or anomalies, there are some indications that extremely high intelligence combined with other factors such as creativity might carry a potential risk for various mental disorders [44]–[46]. In a future survey, it would be interesting to search for correlations between perceived intelligence and deviations from facial symmetry.

If intelligence is judged with an accuracy higher than chance, and if intelligence is suggested to be at least partly heritable, we can then expect that intelligence could be an indicator of underlying genetic fitness with interesting consequences for human evolution [16], [17], [47], [48]. Our results necessarily imply the following question: Why does perceived intelligence reflect measured intelligence in men but not women.

If facial indicators of intelligence are heritable, and if particular genes are not located on the Y sex chromosome, then both sons and daughters of an intelligent-looking father will obtain the alleles for an intelligent-looking face. One possible explanation is that cues of higher intelligence are sexually dimorphic and are thus apparent only in men's faces, e.g. due to some genetic and developmental association to sex steroid hormonal agents during puberty [49]–[51]. If this is true, then the attribution of intelligence in infant faces should not differ between male and female children. When estimating the intelligence of women's faces, observers mechanically use criteria that “work” in men's faces, i.e. the criteria that objectively reflect intelligence in men.

Another option is that women are pervasively judged according to their attractiveness. The strong halo effect of attractiveness may thus prevent an accurate assessment of the intelligence of women. This seems to be supported by a significantly higher correlation of perceived intelligence with attractiveness in women's faces (r = 0.901) in comparison to that in men's faces (r = 0.502).

The third possible explanation is that facial indicators of intelligence are signals rather than cues and that the honest signalling of intelligence is adaptive for men but not for women. It can be speculated, for example, that because of their *mixed mating strategy*, women prefer dominant men as extra-pair sexual partners while at the same time they seek men who are more willing to invest in their offspring as long-term or social partners [52]. It is known that while in the fertile phase of cycle and probably in search of good genes, women prefer creative intelligence to wealth especially in short-term mating [18]. On the other hand, a woman seeking a long term relationship could prefer a less intelligent but honest man, who compensates by long term provisioning, protection and a greater investment in childrearing. At the same time, the prevalence of the mixed mating strategy would influence so as to lead to frequency-dependent selection, and result in the stable coexistence of highly and lowly intelligent men within a population. To test this hypothesis, it will be necessary to search for correlations between women's preferences during their menstrual cycle and the IQ of their preferred partners.

To conclude, humans were able to estimate actual intelligence from facial photographs of men but not women. The attractiveness ratings were not statistically related to measured intelligence in both men and women. No difference between men and women existed in the raters' ability to assess intelligence, and no specific traits that correlated with real intelligence were detected with standard geometric morphometric methods. Men and women with specific facial traits were perceived as highly intelligent. However, these faces of supposed high and low intelligence probably represent nothing more than a cultural stereotype because these morphological traits do not correlate with the real intelligence of the subjects.
